# Half-Curcuminoids Encapsulated in Alginate–Glucosamine Hydrogel Matrices as Bioactive Delivery Systems

**DOI:** 10.3390/gels10060376

**Published:** 2024-05-30

**Authors:** Florentina Monica Raduly, Valentin Raditoiu, Alina Raditoiu, Cristian Andi Nicolae, Maria Grapin, Miruna Silvia Stan, Ionela Cristina Voinea, Raluca-Ioana Vlasceanu, Cristina Doina Nitu, Dan F. Mihailescu, Speranta Avram, Maria Mernea

**Affiliations:** 1 National Research and Development Institute for Chemistry and Petrochemistry—ICECHIM, 202 Splaiul Independentei, 060021 Bucharest, Romania; monica.raduly@icechim.ro (F.M.R.); coloranti@icechim.ro (A.R.); ca_nicolae@yahoo.com (C.A.N.); maria.grapin@icechim.ro (M.G.); 2 Department of Biochemistry and Molecular Biology, Faculty of Biology, University of Bucharest, 91-95 Splaiul Independentei, 050095 Bucharest, Romania; cristina.nica@drd.unibuc.ro (I.C.V.); bizon.raluca_ioana@s.bio.unibuc.ro (R.-I.V.); 3 Department of Anatomy, Animal Physiology and Biophysics, Faculty of Biology, University of Bucharest, 91-95 Splaiul Independentei, 050095 Bucharest, Romania; cristina.nitu@iob.ro (C.D.N.); dan.mihailescu@bio.unibuc.ro (D.F.M.); speranta.avram@bio.unibuc.ro (S.A.); maria.mernea@bio.unibuc.ro (M.M.); 4 Institute of Oncology “Prof. dr. Al. Trestioreanu”, 252, Fundeni, 022328 Bucharest, Romania

**Keywords:** modified curcumins, microwave, bioavailability, antitumor properties, neurological diseases, hydrogel matrices, polysaccharides

## Abstract

The therapeutic effects of curcumin and its derivatives, based on research in recent years, are limited by their low bioavailability. To improve bioavailability and develop the medical field of application, different delivery systems have been developed that are adapted to certain environments or the proposed target type. This study presents some half-curcuminoids prepared by the condensation of acetylacetone with 4-hydroxybenzaldehyde (C1), 4-hydroxy-3-methoxybenzaldehyde (C2), 4-acetamidobenzaldehyde (C3), or 4-diethylaminobenzaldehyde (C4), at microwaves as a simple, solvent-free, and eco-friendly method. The four compounds obtained were characterized in terms of morphostructural and photophysical properties. Following the predictions of theoretical studies on the biological activities related to the molecular structure, in vitro tests were performed for compounds C1–C3 to evaluate the antitumor properties and for C4’s possible applications in the treatment of neurological diseases. The four compounds were encapsulated in two types of hydrogel matrices. First, the alginate–glucosamine network was generated and then the curcumin analogs were loaded (G1, G3, G5–G7, and G9). The second type of hydrogels was obtained by loading the active compound together with the generation of the hydrogel carrier matrices, by simply dissolving (G4 and G10) or by chemically binding half-curcuminoid derivatives to glucosamine (G2 and G8). Thus, two types of curcumin analog delivery systems were obtained, which could be applied in various types of medical treatments.

## 1. Introduction

Diketonic compounds have long been at the attention of researchers, as they are isolated from plants such as turmeric (*Curcuma longa*) or ginger (*Zingiberofficinale*), used in traditional medicine, and recognized for their beneficial effects in treating many diseases [1,2,3,4]. Of these 2,4-pentanedione structures, the derived curcumin has been extensively studied in terms of molecule symmetry and keto-enol and cis-trans isomeric structures [5,6,7,8,9]. Based on these considerations, a series of analogs were developed, such as symmetrical β-diketone derivatives obtained by alkylation, acylation, or complexing with metals, especially rare metals. These compounds have applications in biology, medicine, and catalysis [10,11,12,13,14]. There are recent studies in the literature on the applications of conditioned curcumins as hybrid materials in the field of nonlinear optics, as marking agents for the identification of heavy metals, and as aqueous nitro-derivatives and photocatalysis [15]. Although medical applications remain the most important, they have the most difficult conditions to meet because of the low bioavailability and rapid metabolism of curcumin compounds. The literature demonstrates that curcumin, because of its anti-inflammatory and antioxidant properties, can prevent cancer by suppressing tumor progression [2]. Thus, through its immunomodulatory properties, curcumin interacts with various immune mediators such as cytokines, radical oxygen species (ROS), cyclooxygenase (COX-2), nuclear factor κB (NF-κB), protein kinase B (AKT), protein activator 1 (AP1), signal transducer, and transcription activator 3 (STAT3), which play important roles within the process of cancer initiation and development [16,17,18]. Although a large number of studies show an increased interest in curcumin derivatives, it has not yet been possible to find the ideal structure in terms of biocompatibility and antitumor activity. For this purpose, in parallel with symmetric β-diketone derivatives, asymmetric analogs obtained by synthesis were also studied. Because of the possibilities of functionalization of the diketonic skeleton, the marginal phenyl, and the ketone or the methylene group, the resulting asymmetric compounds have important applications in the medical field, aiming at potentiating the three areas of biological activities [19,20,21,22,23,24]. In recent years, hybrid materials have been studied for applications in the field of biology. The problems that the scientific community in the field must solve are related to the reduced solubility of curcumin derivatives in aqueous media and the delivery of active compounds to the target site without altering their bioactive properties. For this purpose, the grafting of hydrophilic groups on the curcumin skeleton or different conditioning methods in the form of nanoparticles, (nano)emulsions, and hydrogels were investigated.

Alginate is the most versatile polysaccharide matrix used to create bioactive compound delivery systems. Therefore, through different polymerization processes and mechanisms in the presence of cross-linking agents, micro/nano-capsules loaded with antibiotics, and natural or synthetic compounds with antitumor or antimicrobial properties were obtained [25,26,27,28]. These polymer matrices were adapted to the structure of the hosted compounds and the intended application, with the resulting delivery systems having different properties for therapeutic purposes [29,30,31].

In this regard, the current study sought to demonstrate the antiproliferative effect on cervical cancer cells and a decreased toxicity on non-tumoral cells by testing several derivatives of curcumin that were produced using green synthesis. We obtained a series of asymmetric β-diketone compounds through an economical method used in the microwave field. The structures of the compounds were obtained following a condensation reaction, in which a substituted phenyl group forms a new C=C bond with one of the methyl groups of acetylacetone through the aldehyde group. Different groups were grafted on the aromatic ring including hydroxyl (C1), hydroxyl and methoxy (C2) [21,23], acetamide (C3), and diethylamino (C4). The presence of these groups conferred certain characteristics that influence the biochemical properties and antitumor activity of the synthesized compounds. For the obtained compounds, two types of hydrogels were studied as delivery methods. In the G2 and G8 hydrogels, the active compounds were directly linked to the alginate–glucosamine host matrix through Schiff bases, resulting from the reaction between curcumin derivatives and glucosamine. The second type aimed to obtain hydrogels from alginate and glucosamine, in which the active compounds C1–C4 were then loaded, resulting eight composite materials. The obtained composite materials were morpho-structurally characterized in order to study their characteristics, resulting from the interactions established between the host matrix and the hosted compound. These properties are important for the future establishment of potential applications in anticancer therapies, treatments for neural diseases, bandages and regenerative materials, and more.

## 2. Results and Discussion

### 2.1. Synthesis of Asymmetric β-Diketones and Schiff Bases

**Figure 1:**
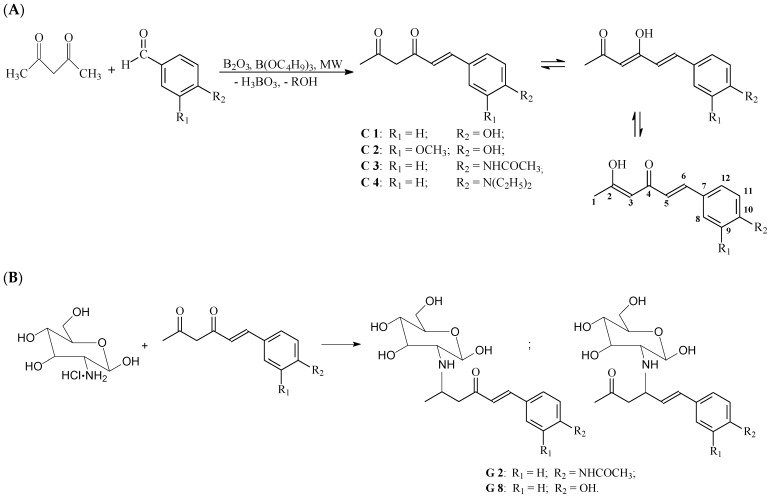

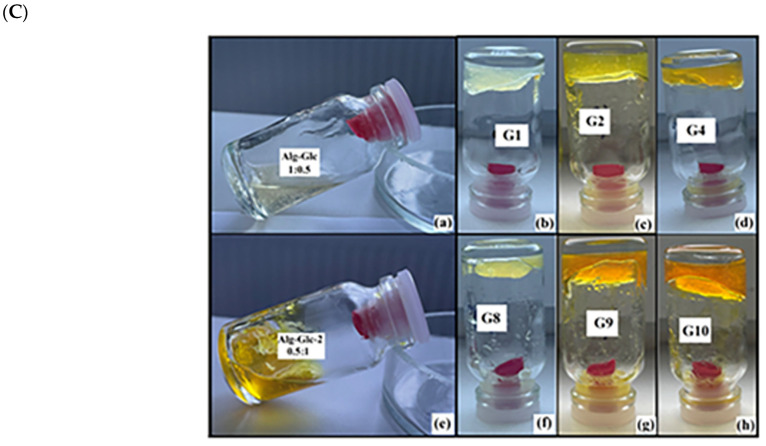
(**A**): The general synthesis of 6-aryl-5-hexene-2,4-diones (C1–C4). (**B**): Synthesis of Schiff bases. (**C**): Hydrogel matrices generate by mixing sodium alginate and glucosamine in different volumetric ratios, Alg:Glc 1:0.5 (a), 1:1 (**b**–**d** and **f**–**h**), 0.5:1 (**e**).

The half-curcuminoid derivatives (C1–C4) were synthesized in a microwave field according to a method developed by us and already presented elsewhere [32]. The curcumin analogs (Figure 1A) were prepared by condensation between acetylacetone and aromatic aldehyde using a simple, solvent-free, and eco-friendly method. Various factors for the condensation reaction were adjusted, including the molar ratio of reactants, microwave power, and irradiation time. The synthesized compounds were purified by recrystallization from ethyl-acetate–methanol = 3:2 (*v*/*v*) and characterized by melting point determination and thin layer chromatography (TLC). Following the development process, retention factor (R_f_) characteristics of the four types of structures were obtained. Compounds C1 and C3 were then used to obtain Schiff bases (Figure 1B) by condensation with the amino group of glucosamine according to Pérez’s method [33] with slight modifications. The method used consisted of obtaining an acidic solution of glucosamine in an ethanol–water mixture over which an alcoholic solution of C1 or C3 dye was added (glucosamine– dye, molar ratio of 4:1). The mixture was heated to 70 °C for 4 h and then cooled and kept in the refrigerator overnight; the resulting precipitate was purified from the ethyl acetate–methanol mixture (5:3, *v*/*v*). In this way, asymmetric β-diketonic structures linked to monosaccharide residues resulted. The progress of the condensation reaction was followed by the TLC method using hexane–acetone–methanol (6:2:1, *v*/*v*) as the eluent on a Silicagel 60 F_254_plastic TLC plate.

### 2.2. Obtaining Glucosamine Alginate Hydrogels Loaded with Asymmetric β-Diketones

The hydrogel matrices were generated by mixing a sodium alginate solution with a glucosamine solution in different volumetric ratios (Figure 1C(a,e)) under an ultrasonic bath. In order to obtain the gelled matrices over the aqueous solution, the glucosamine solution was added in a mass ratio of alginate–glucosamine 1:0.1–0.5 without the gelation process being observed (Figure 1C(a)). With the increase in the amount of glucosamine above these values, the mass of the mixture began to acquire greater viscosity, and after 10 min of ultrasonication, a homogenous gelled and transparent mixture was obtained (Figure 1C(b–d,f–h)). Doubling the amount of glucosamine in relation to alginate led to obtaining gelled agglomerates with different sizes and an inhomogeneous liquid–gel mixture (Figure 1C(e)). In the cases of G4, G5, G9, and G10, when DMSO was used to solubilize the active compounds, the resulting gelled matrices had a more rigid structure than those in which the encapsulated compounds were dissolved in ethanol. The best results were obtained after combining the volumetric ratio of alginate–glucosamine–dye = 1:1:0.5 (Table 1). In order to study the effect of the generated matrix on the hosted dye, two types of hydrogels were made. In the first type, the matrix was generated, and then it was loaded with dye (G1, G3, G5–G7, and G9). The second type of composite was obtained after adding a solution of glucosamine and dye over the sodium alginate (G4 and G10) or a Schiff base solution resulting from the condensation of dyes C1 and C3 at the amino group of glucosamine (G2 and G8). By generating these types of hydrogel matrices, we aimed to obtain delivery systems for compounds derived from curcumin with a hydrophobic character that reduces bioavailability. Furthermore, we tried to improve the hydrophilic properties by directly binding the active compounds (C1 and C3) to the monosaccharide. Hydrogel matrices loaded with active compounds can be used to overcome the dermal barrier for delivery in deep subcutaneous layers both in joint treatments and through intestinal absorption.

### 2.3. Morphostructural Characterization of Half-Curcuminoid Analogs and Hydrogel Matrices by Spectrophotometric Methods

#### 2.3.1. IR Spectroscopy Method

**Figure 2:**
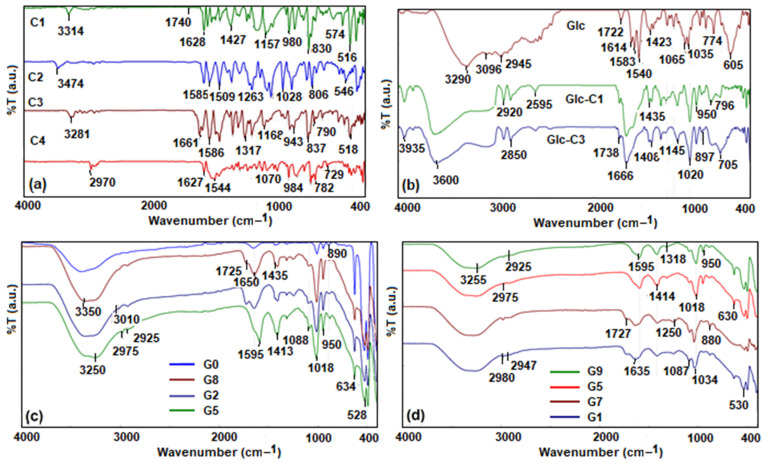
FTIR spectra of asymmetric β-diketones derivatives (**a**), Schiff bases (**b**), and hydrogel matrices loaded with them (**c**,**d**).

The existence of the ketone group was established by infrared spectra, more precisely, by the band situated in the range 1630–1650 cm^−1^ corresponding to the stretching vibration of C=O, which is a characteristic of α- and β-unsaturated carbonyl compounds. The stretching vibration of OH groups involved in hydrogen bonds was located at 3470–3314 cm^−1^, and the aromatic C-H stretching vibration [34,35] was found at 3010–3050 cm^−1^. The asymmetric stretching vibration of the methylene group was situated at 2925–2970 cm^−1^, while the enolic form of the synthesized compounds was supported by the presence of a characteristic band corresponding to the stretching vibration C-OH at 1118–1168 cm^−1^. The functional groups linked on the aromatic rings showed characteristic signals (Figure 2a). The C1 and C2 showed an asymmetric stretching vibration at 1427 and 1417 cm^−1^, respectively, which were attributed to C-O-H (in-plane bending), while the absorption bands around 1263 and 980 cm^−1^ were attributed to the aromatic C-OH stretching vibration and to O-H out-of-plane bending. The absorption band at 3281 cm^−1^ was attributed to the stretching vibration of N-H, while at 1661 and 1638 cm^−1^, the first band and the second band characteristics, respectively, of the amide were found. The spectra of compounds C3 and C4 show characteristic absorption bands around 1266–1296 cm^−1^ ascribed to the stretching vibration of C_aromatic_-N, while the second absorption band around 1185–1168 cm^−1^ corresponded to the stretching vibration of C_aliphatic_-N. The presence of Schiff bases is confirmed in the FTIR spectra shown in Figure 2b by the presence of broad bands at 3600 cm^−1^, attributed to the vibrations of the N-H and O-H bonds. The increase in intensity and the displacement of the bands at 2900–2850 cm^−1^ compared with those of the glucosamine spectrum and the presence of bands centered at 1666 cm^−1^ are characteristic of the enamine functionality [36].

These compounds directly linked to monosaccharide residues together with alginate formed structures similar to the matrix obtained without the hosted compound (Figure 2c), with the IR spectra differing only in the range 1700–1080 cm^−1^. Thus, the band at 1725 cm^−1^ was attributed to the stretching vibrations of the C=O bonds, while the band at 1650 cm^−1^ was characteristic of the vibrations of the water bond vibration and overlapped with the stretching vibrations of the bonds of the amide groups. This was confirmed by comparing the spectra with that of the G5 composite, in which the dye is not linked to the monosaccharide unit and the band undergoes a shift to 1595 cm^−1^, assigned to the vibrations of the N-H bond in the glucosamine structure. As can be seen in Figure 2d, in the obtained composites, the presence of the dye with different auxochromes does not influence the FTIR spectra (G1 and G7 or G5 and G9). They are different because of the sequestered solvents (water, ethanol, or DMSO) in the hydrogel network that cause the displacement of the vibration bands characteristic of C-H bonds from 2975 to 2925 cm^−1^, the band in the area 1595–1635 cm^−1^, characteristic of the vibrations of O-H and N-H bonds, or the band ascribed to C-O-C bond vibrations in the range 1018–1034 cm^−1^.

#### 2.3.2. SEM-EDX Analysis

**Figure 3:**
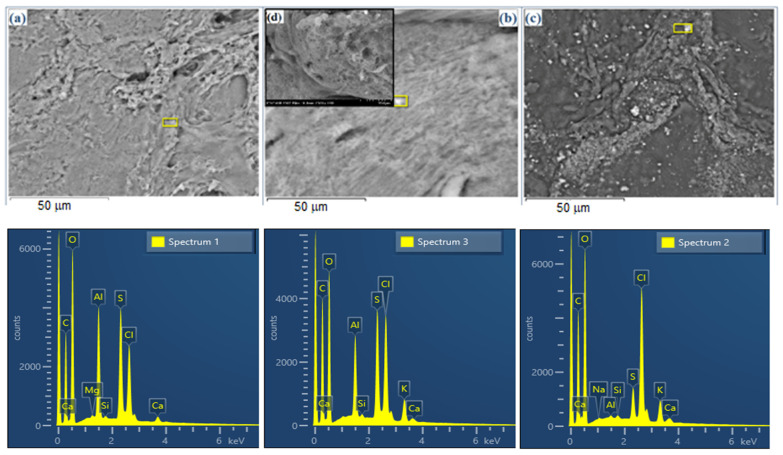
SEM images and the EDX spectra of the carrier host matrix (blank) (**a**); the hydrogel matrix loaded with C2 in the generation stage of the matrix (**b**,**d**) and the hydrogel matrix loaded with C2 in the subsequent stage of the generation of the carrier matrix (**c**).

The method of obtaining the hydrogels determines the architecture of the gelled network, as can be observed in the SEM images (Figure 3a–d). The recorded images show the hydrogelated host matrix (Figure 3a) with a certain structure, with voids of different sizes, in which the solutions of the curcuminic compounds are loaded (Figure 3c). In contrast to these, Figure 3b,d show the gels in which the dye to be encapsulated is introduced at the beginning of the gelation process by simply dissolving it in a mixture with glucosamine or by directly binding to the monosaccharide. Thus, the obtained composites have a homogeneous structure, and the dye is evenly distributed in the carrier network.

Examining the results of the EDX analyses for the hydrogel matrices loaded with curcumin halves, it appears that their structural properties directly influence the amount of active compounds retained in the gel network. Thus, the increase in the molecular mass of the curcuminic derivatives in conjunction with the volume of the auxochrome groups and their hydrophobic properties, lead to a decrease in the quantity encapsulated. At the same time, the structure of the curcuminic derivatives and the intermolecular interactions with natural polymers lead to gelled networks with different architectures (Figures S1 and S2). Through the EDX analysis and by comparing the data from Table 2, it can be observed that the loading of the matrices with active compounds is significantly affected by the method of obtaining the composites. The generation of the carrier matrix and its loading in the later stage (G4) leads to a difference of 6.8% carbon, compared with the initial matrix, which is about 3% compared with G5. However, in the case of G4, the values are not relevant because of the inhomogeneity in the composite, where the cages formed in the gelled network favor the uneven storage of the dye in the network.

#### 2.3.3. UV Absorption Spectra

**Figure 4:**
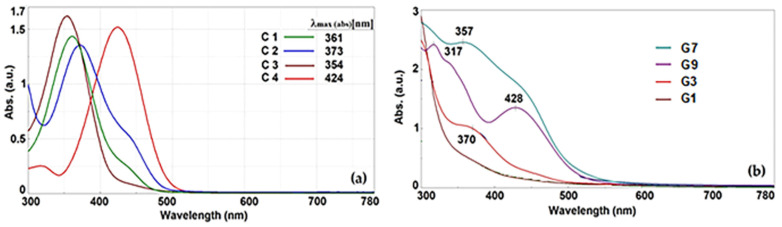
Absorption of asymmetric β-diketone derivative (in methanol, 2 × 10^−4^ mol/L) (**a**) and the hydrogel matrices loaded with them (**b**).

There are studies [37] that confirm the conformational stability of β-diketones in cis-enol forms because of an intramolecular hydrogen bond system, which forms a six-member ring. For the asymmetric di-ketones studied by us, the electron effects of auxochromes belonging to phenyl groups influence keto-enol tautomerism and cis-trans isomerism. All these tautomeric states and their interactions with the polar solvent directly influence the spectrum absorption and fluorescence emission properties. Thus, it was observed that because of a lower conjugation on the molecule, the position of absorption maxima of asymmetric compounds (Figure 4a) was displaced hypsochromic with 52–70 nm compared with symmetrical fluorophores [32].

The UV absorption spectra, recorded in methanol, show that for all the compounds, the transition corresponding to π ‒ π* (due to the chromophore C=C) and n ‒ π* (due to the chromophore C=O) are overlapped and bathochromic-shifted in the range 300–450 nm. The maximum adsorption of compound C1 was measured at 361 nm. The maximum absorption peaks were strongly influenced by the aryl groups’ conjugation with auxochromes that are characterized by low levels of the extinction coefficient. The presence of the methoxy group on the aromatic ring leads to a bathochromic effect of 12 nm compared with C1, while the diethylamino group present in compound C4, through its electron-donating effect, causes a much higher bathochromic displacement of about 63 nm. The UV absorption bands are broad and indicate the presence of more than one isomeric form in the ground state. Based on this finding, the important role of auxochromes regarding the shape of the absorption bands and other spectral properties of compounds was established. The presence of hydroxyl, methoxy, or amino electron-donating auxochromes results in an increase in electronic density both on the aromatic ring and on the entire structure of the dyestuff molecules, thus causing a bathochromic displacement of the absorption band.

Hydrogels by their nature are defined as biphasic materials with a high water content. Starting from this, it was expected that the absorption spectra of the composite materials loaded with curcumin derivatives would be directly influenced by the composition of the interstitial liquid and its interactions with the hosted dye. Thus, in the water/ethanol solvent mixture, the polarity of the environment is lower, and the interactions with the dye take place through hydrogen bonds and polar bonds and lead to broad absorption bands (G1, G3, and G7). In the case of the G9 composite, which contains a water/DMSO mixture with higher polarity, the interaction with C4 leads to the retention of the C4 characteristic absorption band but slightly redshifted (Figure 4b).

#### 2.3.4. Fluorescence Spectra

**Figure 5:**
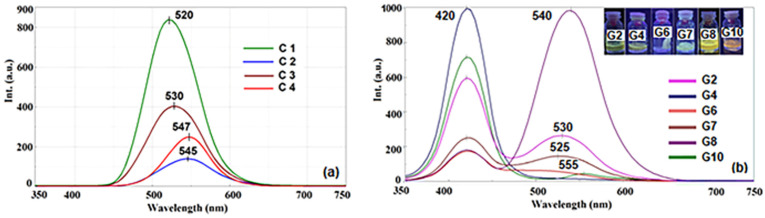
Fluorescence spectra of asymmetric β-diketones derivatives (in methanol, 3 × 10^−5^ mol/L) (**a**) and the hydrogel matrices loaded with them (**b**).

Regarding the fundamentals of the series, it was found that acetylation produced a Stokes shift of roughly 176 nm, which can only be accounted for by a significant alteration in the dyestuff’s dipolar structure upon excitation. The fluorescence spectra (Figure 5a) were measured on alcoholic samples (λ_ex._ = 420 nm), and the highest intensities were recorded for derivatives C1 at 520 nm (Stokes shift Δ~159 nm) and C3 at 530 nm (Stokes shift Δ~176 nm). In good agreement with the literature [37], it was noticed that the fluorescence emission spectra were influenced by the hydrogen bonds established between the protic solvent and the dyes, by the type of auxochromes grafted on the aromatic ring. Thus, in a polar solvent, β-diketone derivatives are predominantly cis-enol or trans-enol forming intermolecular H bonds with the solvent. However, the presence of OCH_3_ in the phenolic fragment has a general action of inhibiting the intensity of fluorescence [32,37]. Figure 4a shows that compound C3 without hydroxyl groups has considerably lower fluorescence intensity than that of compound C1. This phenomenon occurs because of the amide group in the aromatic structure, which has a greater fluorescence quenching effect than the hydroxyl group, because of the hydrogen bonds established with the solvent and the polar tautomeric structures formed. On the other hand, the decrease in fluorescence intensity of the following two compounds, C4 (λ_em_ = 547 nm Stokes shift Δ~123 nm) and C2 (λ_em_ = 545 nm Stokes shift Δ~172 nm), is due to the processes of extinguishing fluorescence resulting from the presence of several stable tautomeric polar structures following light excitation processes. The intensities of the fluorescence spectra of asymmetric compounds synthesized in the present work are much lower than the fluorescence emission of the symmetric analogs. Therefore, the fluorescence properties are influenced by the symmetry in the molecular structure and auxochrome groups grafted on the aromatic ring that establish inter-molecular H-bonds with the solvent molecules.

Moreover, the hosting of active compounds in hydrogel matrices led to obtaining materials with fluorescent properties (Figure 5b), directed by the interaction between the host matrix and the hosted half-curcuminoid compounds. Similar to symmetric compounds [38] loaded on different types of carrier matrices, the asymmetric β-diketonic derivatives encapsulated in the hydrogel matrices present two fluorescence emission maxima. The first emission band at 420 nm is attributed to the chromophore and is common to the obtained composites, while the second emission band is due to the interactions established between the auxochromes grafted on the aromatic ring and functional groups of the carrier matrix. Therefore, it can be observed that in the case of G4 and G10, which contain a polar water/DMSO mixture as an interstitial liquid, the second fluorescence emission band is of low intensity. This is due to the hydrogen bonds established between the hydroxy and methoxy groups at G4, the diethyleneamino group at G10, or even the intermolecular bonds with the hydroxyl groups of the host matrix, all of them being fluorescence quenching pathways. In the case of composites G6 and G7, a decrease in fluorescence is observed because of fluorescence quenching attributed to the aggregation processes of C2 and C3 hosted in the cages of the hydrogel network, as confirmed by the SEM images (Figure 3c). The most red-shifted intense fluorescence emission band was recorded in the case of the G8 composite, which contains C3 luminophore, characterized by the presence of bulky substituents that prevent interactions between fluorophore and environments.

#### 2.3.5. DSC Analyses

**Figure 6:**
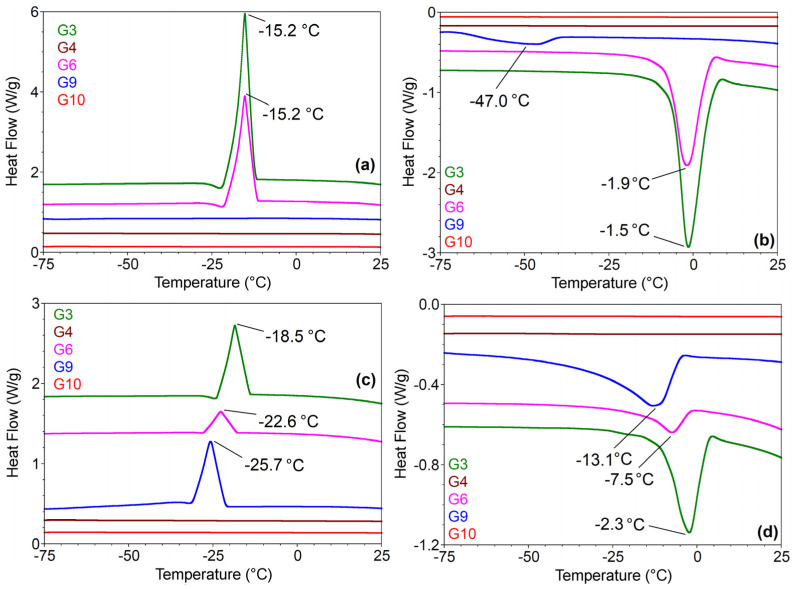
DSC first heating (**a**) and cooling (**b**) curves and second heating (**c**) and cooling (**d**) curves of hydrogels loaded with half-curcuminoid derivatives.

Because of the differences in the morpho-structural properties of the two types of composites, it was considered necessary to evaluate the materials from the point of view of thermal stability over time (Figure 6) through Differential Scanning Calorimetry Techniques (DSC). In the first cooling scan, materials G4, G9, and G10 did not show kinetic events or phase transformations (Figure 6a). These characteristics are preserved by G4- and G10-gelled materials during the first heating scan and during the second heating–cooling cycle to which they are subjected. The results confirm once again that the addition of the active compound at the beginning of the gelation process leads to homogeneous and much more stable materials. The composite materials obtained by the initial formation of the carrier matrix followed by loading it with the active compounds (G3, G6, and G9) lead to irregular networks, as observed in the SEM images (Figure 3a), characterized by the accumulation of dye and interstitial solvent in the cells of the gelled network. DSC analyses show crystallization phenomena with freezing points characteristic of mixtures of solvents sequestered in the polysaccharide network (Figure 6a,c). In the second heating cycle, the diagrams show the maxima shifted compared with the first cycle, which may be due to the rearrangement of the solvent and dye molecules or the superficial modification of the gel matrix because of the increase in the volume of the interstitial mixture upon freezing [39,40,41]. Melting temperatures vary for each type of gelled material depending on the mixture of solvents, ethanol/water in G3, water in G6, and DMSO/water in G9 (Figure 6b,d).

### 2.4. Computational Analysis of Half-Curcuminoid Derivatives

In silico predictions are used to filter and prioritize potential drug candidates. Several models are used to predict the drug-likeness of compounds, their bioavailability, ADMET features, and targets. We performed this initial stage to show that the compounds present promising pharmacokinetic and pharmacodynamic properties, which support their further testing in in vitro conditions. The only aspect that we could address experimentally is the antitumoral effect of the compounds predicted based on the indications of their possible targets. The experiments addressed the cytotoxicity of the compounds in the HeLa cell line. All compounds were predicted to have antitumoral properties; therefore, they were tested in vitro. Following the experiments on the HeLa cells, the cytotoxic activities were confirmed only for compounds C1–C3. In the case of compound C4, we showed that it has no inhibitory effect on the proliferation of HeLa cells. This does not exclude the possibility that it shows antitumoral properties on other cancer cell lines.

The pharmacological properties of the obtained compounds were evaluated theoretically in relation to their structural formulas [42,43], using online platforms for predicting pharmacokinetic [34] and pharmacodynamics properties [35,44].

#### 2.4.1. Drug-Likeness Assessment

The variation in compound properties due to changes in substituents, as well as between the keto and enol forms, can be seen in Table S1 (Supplementary Materials). According to LogP values, the C1, C2, and C3 compounds are slightly lipophilic, with C4 being more lipophilic. Also, the C4 compound presents the lowest number of hydrogen bond acceptors and donors, but it is the most flexible, presenting the highest number of rotatable bonds.

The drug-likeness assessment, as supported by results presented in Table S2 (Supplementary Materials), show that compounds C1–C4 comply without any violation of the rules of Lipinski [45,46], Veber [47], Ghose [48], Egan [49], or Muegge [50]. These rules state the optimal ranges of physicochemical parameters of molecules that are likely to act as drugs. In addition, the compounds show good bioavailability scores of 0.55 (keto forms) and 0.85 (enol forms), meaning that their probabilities to present at least 10% oral bioavailability or measurable Caco-2 permeability are 55% and 85%, respectively [43]. These results suggest that compounds C1–C4 could represent therapeutic agents. The same predictions were made for the two Schiff bases of C3 (G2) and C1 (G8). These compounds are more hydrophilic than their parents, presenting larger numbers of hydrogen bond donors and acceptors and also larger numbers of rotatable bonds. G2 and G8 comply with the drug-likeness rule of Lipinski but present one violation of the other rules. Their bioavailability scores are 0.55, being equal to the bioavailability of their parent compounds in the keto form, but lower than their parent compounds in the enol form.

#### 2.4.2. Predicted Drug Disposition Properties

Table S3 (Supplementary Materials) displays the substances’ estimated absorptions, distributions, and toxicity properties. The calculated absorption properties, reflected by Caco2 permeability and intestinal absorption parameters, reveal that compounds C1–C4 should present a high gastrointestinal absorption. Compounds G2 and G8 present low Caco2 permeability and intestinal absorption percentages close to the threshold for poorly absorbed compounds (intestinal absorption < 30%). All compounds except for C4 present high skin permeability. The skin permeability of C4, especially in enol form, is close to the threshold value for compounds that have fair skin permeability. In regard to their distribution, the analysis of the predicted values for the blood–brain barrier (BBB) and central nervous system (CNS) permeabilities shows that compounds C1-C4 could penetrate the BBB to be distributed into the brain. The best values were obtained in the case of compound C4, in both keto and enolic forms. These values point toward potential applications of the compounds in the central nervous system. LogBB and LogPS values predicted for compounds G2 and G8 suggest a poor distribution to the CNS. The predicted toxicity parameters of all the compounds suggest that they are non-toxic for the heart (not inhibitors of hERG I or II potassium channels) and have no mutagenic potential (no AMES toxicity). An exception is the keto form of compound C3, which presents AMES toxicity. Compounds C1-C4 and G8 were predicted to be non-toxic for the liver (no hepatotoxicity), but G2 might present hepatotoxicity.

#### 2.4.3. Predicted Pharmacodynamic Features

The molecular targets of the C1–C4, G2, and G8 molecules were assessed using SwissTarget [35]. The platform predicted that the compounds have a low probability of modulating different targets in the case of C1 in enol form and compounds C3, C4, G2, and G8. Keto C2 was predicted to present a 52% probability of modulating the beta amyloid A4 protein (APP), a 35% probability of modulating monoamino oxidase A (MAOA), and a 28% probability of modulating beta-secretase 1 (BACE1). C2 in enol form was predicted to modulate APP with a probability of 32%. C1 compound in keto form was predicted to modulate beta-secretase 1 with a probability of 21%. It is interesting to note that APP and BACE1 are two relevant targets for Alzheimer’s disease [51,52,53], while MAOA is a target for antidepressant drugs [54,55]. The previously described beneficial effects of the parent compound curcumin in Alzheimer’s disease [56], in particular, and in brain diseases [57], in general, support these predicted effects.

In addition to these results, we used Super-PRED Target Prediction [44] to evaluate the compounds. The platform identifies molecular targets of compounds and predicts their probability percentage. In addition, it detects the indications of predicted targets that are ranked by their probability scores. The platform predicted several targets for all compounds (C1–C4, G2, and G8). From these, we retained two common high probability (>80%) targets of all compounds in both keto and enolic form, namely, DNA (apurinic or apyrimidinic site) lyase and DNA topoisomerase II alpha. The platform reports these two proteins to be involved in solid tumors and different types of cancers with a high probability. Again, these results are expected since curcumin, the parent compound for the derivatives presented here, was shown to inhibit DNA (apurinic or apyrimidinic site) lyase (also known as Apurinic/apyrimidinic end nuclease 1) resulting in lymphoma remission [58], and to target DNA topoisomerase II resulting in cancer cells [59].

The predicted targets of the compounds suggest their usefulness in two therapeutic directions, namely, in the treatment of conditions that affect the CNS, like Alzheimer’s disease, and of solid cancers of various types. These two indications of the compounds are not surprising as the two diseases lie at opposite ends of the cell division spectrum and share important common features [60]. In the case of C1 in enol form and G2, we also identified targets important in inflammation. These are estrogen receptor beta (C1 in enol form), formyl peptide receptor 1 (G8 and G2), cyclin-dependent kinase 1/cyclin B1, and PI3-kinase p110-gamma subunit (G2). These predictions point to an additional therapeutic application of the compounds, namely, in inflammation and inflammation-related diseases. This direction is in agreement with the known beneficial effect of curcumin in inflammation [61].

In view of the predictions that the analyzed compounds could be beneficial in cancer, their activity was evaluated in vitro. As further presented, we tested their cytotoxic effect on a cervical carcinoma cell line (HeLa cells), as well as on a normal cell line (MRC-5 cells).

### 2.5. Biological Evaluation

To study the level of cell viability, MTT and Griess tests were used. The exposure of human cells to different concentrations of curcumin derivatives lasted up to 72 h, and the results were analyzed to select concentrations that decreased the viability of tumor cells and did not significantly affect non-tumoral cells.

**Figure 7:**
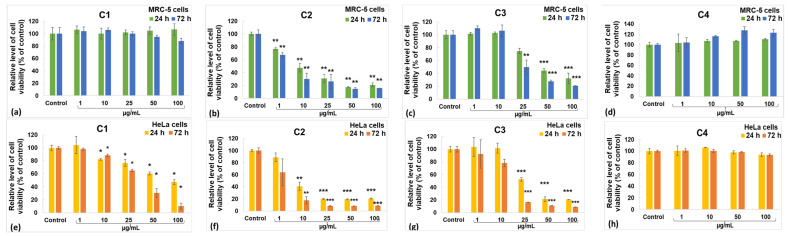
Viability of MRC-5 non-tumoral cells (**a**–**d**) and HeLa cancer cells (**e**–**h**) after 24 and 72 h incubations with the C1 (**a**,**e**), C2 (**b**,f), C3 (**c**,**g**), and C4 (**d**,**h**) curcumin derivatives measured by the MTT assay. Data are expressed as mean ± standard deviation (SD) (*n* = 3) and represented relative to the control (untreated cells). * *p* < 0.05, ** *p* < 0.01 and *** *p* < 0.001 compared with the control.

**Figure 8:**
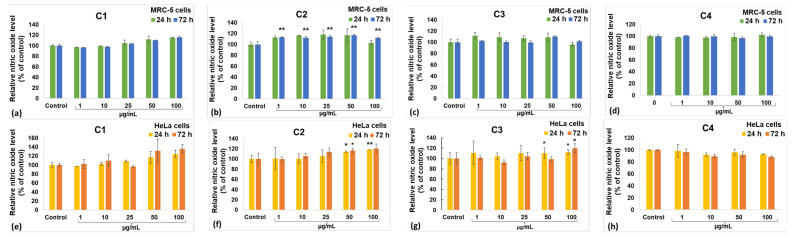
Nitric oxide level released by MRC-5 non-tumoral cells (**a**–**d**) and HeLa cancer cells (**e**–**h**) after 24 and 72 h incubations with the C1 (**a**,**e**), C2 (**b**,**f**), C3 (**c**,**g**) and C4 (**d**,**h**) curcumin derivatives. Data are calculated as mean ± standard deviation (SD) (*n* = 3) and represented relative to the control (untreated cells). * *p* < 0.05 and ** *p* < 0.01 compared with the control.

The MTT test gives data on cell proliferation, survival rate, and metabolic activity. The impact of the incubation of the MRC-5 and HeLa cell lines with diverse concentrations of curcumin derivatives is illustrated in Figure 7. Within the case of the C1 derivative, no noteworthy changes were observed in the MRC-5 cells (Figure 7a), but a dose-dependent diminish was recorded for HeLa cancer cells (Figure 7b). In contrast, doses over 10 µg/mL of C2 induced a significant decrease in the viability of both tumor and non-tumor cells compared with the unexposed control (Figure 7c,d). The incubation with concentrations up to 10 µg/mL of C3 derivative did not modify the viability of MRC-5 cells compared with the control, but it decreased the number of HeLa cells. However, higher concentrations of C3 determined a diminution in viable cells by more than 50% of the control levels for both lines. The capacity to induce apoptosis of cervix cancer cells was also previously described for other curcumin derivatives [62,63]. However, the C4 derivative was not able to decrease the viability of HeLa cells compared with the unexposed control cells (Figure 7h). The level of nitric oxide (NO) released in the culture media (Figure 8) indicates the extent of cytotoxicity caused by curcumin, serving as a marker for inflammatory processes. The C1 derivative did not elevate NO release in the MRC-5 or HeLa cell culture medium compared with the unexposed control cells at concentrations below 50 µg/mL (Figure 8a,b). However, at the 50 µg/mL concentration, there was an increase in NO levels by 10% in MRC-5 cells and by 31% in HeLa cells compared with the control after 72 h. This variation between the two cell types is consistent with the MTT assay findings. The incubation with derivates C2 or C3 did not modify the NO levels much, where the increase with 20% above the control of MRC-5 or HeLa cells was the maximum (Figure 8c–f). Furthermore, no differences compared with the control were noticed after incubation with C4, regardless the cellular type (Figure 8g,h).

**Figure 9:**
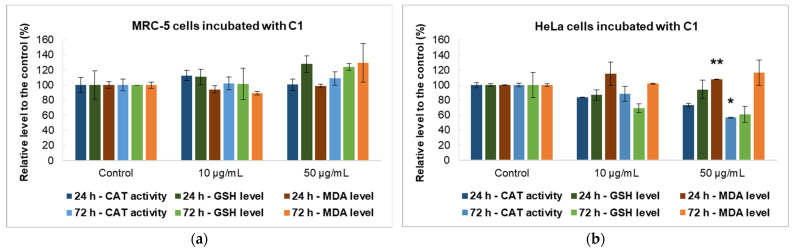
Levels of catalase activity and reduced glutathione and malondialdehyde in MRC-5 non-tumoral cells (**a**) and HeLa cancer cells (**b**) incubated with 10 and 50 μg/mL of the C1 curcumin derivative. Data are calculated as mean ± standard deviation (SD) (*n* = 3) and represented relative to the control (untreated cells). * *p* < 0.05 and ** *p* < 0.01 compared with the control.

Given that the MTT test revealed a significantly greater percentage decrease in cell viability of the cancer cells when compared with non-tumoral fibroblasts, the C1 curcumin derivative was chosen for additional biochemical analysis. This analysis included measuring the levels of malonaldehyde (MDA) and glutathione (GSH), as well as catalase (CAT) activity in both cell types (Figure 9).

CAT is an enzyme that converts harmful hydrogen peroxide into oxygen and water, thus preventing damage to biomolecules. While MRC-5 cells showed no significant changes, there was a notable impact on HeLa cells after 72 h. The activity of CAT decreased significantly (by 43% compared with the control) in HeLa cells when exposed to a concentration of 50 µg/mL C1 during incubation.

GSH, the primary antioxidant in human cells, can interact with xenobiotics directly or by using enzyme-mediated processes. GSH levels increase in cancer cells to protect them from toxins, peroxides, and free radical damage while also maintaining the cellular redox balance. Cells treated with the C1 curcumin derivative did not modify the GSH concentration in MRC-5 cells but decreased the level of this antioxidant in HeLa cells compared with the control. After 72 h of incubation with 50 µg/mL C1, there was a 40% reduction in GSH levels compared with the control. This indicates a change in antioxidant defense mechanisms, leading to the exposure of the cells to oxidative stress and potential cell death.

One of the end-products of polyunsaturated fatty acid peroxidation is MDA. Since free radicals initiate the process of lipid peroxidation in cells, an increase in their level leads to an excess of MDA production. The levels of MDA in patients with cancer are a measure of their antioxidant status and oxidative stress [64]. When MRC-5 cells were exposed to 50 µg/mL of C1 for 72 h, the MDA level increased by 30% compared with the untreated control cells. In HeLa tumor cells, a smaller amount of MDA was reported after exposure to the same concentration of C1 (a 16% increase over control at 72 h). In any case, the MDA level did not increase significantly compared with the control, suggesting that this compound’s activity does not specifically induce lipid peroxidation, at least not for the concentrations and intervals of exposure that were tested.

## 3. Conclusions

Four curcumin-like compounds with various auxochromes grafted onto the aromatic ring were synthesized by a green method. The compounds were morpho-structurally characterized, and their bioactivity was theoretically and in vitro evaluated.

The compounds analyzed present good drug-likeness and bioavailability features. Additionally, they present promising ADMET features, being readily absorbed in the gastrointestinal tract and distributed to the brain, while being non-mutagenic and safe for the heart and liver. The predicted targets of the compounds suggest their utility in three therapeutic directions, namely, Alzheimer disease and other CNS disorders, different types of solid tumors and cancers, and inflammation. Under these conditions, we decided to develop studies on the anticancer activities of compounds C1–C3 and C4 to evaluate their future for applications in the treatment of neurodegenerative diseases. In vitro evaluation of the compounds showed that the C1 compound had cytotoxic impacts on HeLa tumor cells in low concentrations, suggesting noteworthy antitumor properties without influencing normal cells. With these antioxidant and antitumor properties, there are high prospects for future in vivo testing and, along these lines, utilization within the generation of drugs with a focus on anticancer effects. The bioactive properties of curcumin derivatives are overshadowed by their low light stability and low hydrophilicity. Consequently, we considered it appropriate to load the proven active compounds (C1–C3) in hydrogel matrices to eliminate these inconveniences. The stability of hydrogel materials and their behavior depending on temperature define the future medical applications of active compounds with antitumor or neurodegenerative properties. The physical and structural characteristics of the host materials are important in understanding the interaction between the host and the hosted active compound.

## 4. Materials and Methods

### 4.1. Raw Materials for the Synthesis of Beta-Diketone Derivatives and Hybrid Materials

The precursors used for the synthesis and characterization of the β-diketone derivatives were of laboratory reagent grade and were bought from providers such as Merck/Sigma-Aldrich (Darmstadt, Germany). Sodium alginate and glucosamine sulfate were purchased from a local supermarket. The raw materials were used for the synthesis of β-diketone derivatives and hybrid materials without further purification.

### 4.2. Methods of Obtaining Biologically Active Compounds and the Carrier Matrix

#### 4.2.1. Synthesis Methods of the Asymmetric β-Diketone Compounds (C1–C4) and Schiff Bases (Glc-C1 and Glc-C3)

A mixture of acetylacetone 0.41 mL (4 mmol), B_2_O_3_ 0.28 g (0.4 mmol), and tributyl borate 8.6 mL (3.2 mmol) was mixed into a porcelain capsule and irradiated in a microwave field (300 W power, 10 min). The aromatic aldehyde (4 mmol) and dodecylamine (0.162 mmol) were added into the boronic complex of acetylacetonate that resulted after irradiation, and the mixture was further irradiated at microwaves for 5–10 min at 100–300 W. Synthesis reactions were conducted in a microwave kitchen range PLATINUM (Power—1000 W; Frequency—2.45 GHz; Volume—31 L, multimode cavity), where the control of the microwaves was balanced by alterations of the irradiation time and microwaves control (concurring to the sort of fragrant aldehyde utilized). The response blend was evacuated from the stove and cooled to 25 °C. It was dissolved in methanol and precipitated with a 10% acetic acid solution. The solid from the final reaction mass was isolated by filtration and washed with water several times. The solid product was dried at 105 °C. The crude products were recrystallized from ethyl acetate–methanol = 3:2 (*v*/*v*), and after crystallization, the pure products were obtained with yields of 64–82%.

6-(4-hydroxyphenyl)-5-hexene-2,4-dione (C1): Yellow powder, 75% yield. mp 121–125 °C, R_f_ value (TLC): 0.48, Yellow Spot color (UV-365 nm). ^1^H NMR (400 MHz) CDCl_3_. δ, ppm: 7.57 (d, *J* = 10.9, 2H, H-8, H-12), 7.44 (d, *J* = 3.4, 2H, H-9, H-11), 6.85 (d, *J* = 8.5, 1H, H-6), 6.35 (d, *J* = 15.8, 1H, H-5), 5.62 (s, 1H, H-3), 3.81 (s, 2H, H-1), 15.54 (s, 1H, OH’), 15.44 (s, 1H, para-OH), 5.28 (s, 1H, H-3), 2.16 (s, 3H, H-1) confirms the presence of the diketotautomer (Figure 1) [65]. ^13^C NMR (100 MHz) CDCl_3_. δ, ppm: 140.63 (C-10), 134.97 (C-9), 130.10 (C-11), 128.92 (C-8), 128.11 (C-12), 124.04 (C-7), 101.80 (C-5, C-6), 183.30 (C-2). GC-MS (*m*/*z*) 204 M (29%), 147 ^+.^CH_2_COCH_3_ (100%). Anal. Calcd. for C_12_H_12_O_3_ (%): C, 70.59; H, 5.88; O, 23.53. Found (%): C, 70.68; H, 5.84; O, 23.48.

6-(4-hydroxy-3-methoxyphenyl)-5-hexene-2,4-dione (C2): Orange powder, 77% yield. mp 163–165 °C, R_f_ value (TLC): 0.45, Yellow Spot color (UV-365 nm).^1^H NMR (400 MHz), CDCl_3_.δ, ppm: 7.61 (d, *J* = 10.9, 1H, H-8), 7.13 (dd, *J* = 15.8, 1H, H-12), 7.11 (d, *J* = 7.7, 1H, H-11), 6.94 (d, 1H, H-6), 6.50 (d, 1H, H-5), 5.80 (s, 1H, H-3), 3.95 (m, 5H, H-1, meta-OCH_3_), 15.57 (s, 1H, OH’), 6.94 (d, 1H, H-6), 5.85 (s, 1H, H-3) due to the presence of the diketotautomer (Figure 1). ^13^C NMR (100 MHz), CDCl_3_. δ, ppm: 147.84 (C-9), 146.77 (C-10), 140.54 (C11), 127.69 (C-7), 122.88 (C-12), 121.77 (C-8), 101.18 (C-5, C-6), 183.26 (C-2), 55.96 (OCH_3_). GC-MS (*m*/*z*) 234 M (45%), 43 ^+.^COCH_3_ (100%). Anal. Calcd. for C_13_H_14_O_4_(%): C, 66.67; H, 5.98; O, 27.35. Found (%): C, 66.87; H, 5.79; O, 27.34.

6-(4-acetamidephenyl)-5-hexene-2,4-dione (C3): Dark orange powder, 82% yield. mp 158–160 °C, R_f_ value (TLC):0.63, Orange Spot color (UV-365 nm). ^1^H NMR (400 MHz), CDCl_3_. δ, ppm: 7.58 (d, *J* = 7.8, 2H, H-8, H-12), 7.52–7.46 (m, *J* = 7.6, 2H, H-9, H-11), 6.41 (d, 1H, H-6), 6.37 (s, 1H, H-5), 5.64 (s, 1H, H-3), 2.20 (d, *J* = 1.9, 3H, NHCOCH_3_), 2.17 (s, 4H, NHCOCH_3_), 15.46 (s, 1H, OH’). ^13^C NMR (100 MHz), CDCl_3_. δ, ppm: 139.46 (C-8), 139.08 (C-12), 131.14 (C-9), 130.90 (C-11), 128.81 (C-10), 121.66 (C-7), 101.18 (C-5, C-6), 197.70 (C-3), 177.15 (C-2), 31.90 (C-1), 168.44 (NHCOCH_3_), 14.13 (NHCOCH_3_).GC-MS (*m*/*z*) 245 M (17%), 43 ^+.^COCH_3_ (100%). Anal. Calcd. For C_14_H_15_NO_3_(%): C, 68.57; H, 6.12; N, 5.71; O, 19.59. Found (%): C, 68.63; H, 6.28; N, 5.54; O, 19.55.

6-(4-diethylaminophenyl)-5-hexene-2,4-dione (C4): Red powder, 64% yield. mp 112–116 °C, R_f_ value (TLC): 0.39, Orange Spot color (UV-365 nm). ^1^H NMR (400 MHz), CDCl_3_. δ, ppm: 7.57 (d, *J* = 7.8, 2H, H-8, H-12), 7.53 (m, *J* = 7.6, 2H, H-9, H-11), 6.67 (d, *J* = 8.3, 2H, H-6), 6.29 (d, *J* = 8.3, 2H, H-5), 5.62 (d, 1H, H-3), 3.42 (d, *J* = 7.1, 4H, H-1), 2.15 (m, 3H, N(CH_2_CH_3_)_2_), 1.57 (s, 4H, N(CH_2_CH_3_)_2_), 15.69 (s, 1H, OH’), 6.28 (d, 2H, H-5), 5.59 (d, 1H, H-3), 3.41 (d, 4H, H-1) due to the presence of the diketotautomer (Figure 1). ^13^C NMR (100 MHz) CDCl_3_. δ, ppm: 146.98 (C-10), 130.52 (C-8), 130.25 (C-12), 129.90 (C-9), 129.62 (C-11), 122.60 (C-7), 101.29 (C-5, C-6), 138.33 (C-4), 197.70(C-3), 176.80 (C-2), 43.92 (N(CH_2_CH_3_)_2_), 29.71 (C-1), 27.08 (N(CH_2_CH_3_)_2_). GC-MS (*m*/*z*) 259 M (48%), 244 M-CH_3_ (100%). Anal. Calcd. for C_16_H_21_NO_2_(%): C, 74.13; H, 8.11; N, 5.41; O, 12.36. Found (%): C, 74.19; H, 8.12; N, 5.18; O, 12.51 (see Supplementary Materials, S2).

In 10 mL of an ethanol–water mixture (50%), 0.2 g of glucosamine sulfate was dissolved, to which 0.5 mL of 2 M hydrochloric acid and an alcoholic solution of C1 or C3 dye concentration 0.2% (glucosamine: dye, molar ratio of 4:0.5) was added. The mixture was heated at 70 °C for 4 h and then cooled and refrigerated overnight. The resulting light-yellow precipitate was purified from an ethyl acetate–methanol (5:3, *v*/*v*) mixture. In this way, asymmetric β-diketonic structures linked to monosaccharide resulted. The progress of the condensation reaction was followed by the TLC method using hexane–acetone–methanol (6:2:1, *v*/*v*) as an eluent on a SilicagelMerck 60 F_254_plastic TLC plate (R_f_ = 0.4). Individual spots were identified by visually observing colored spots using coordinate visualization under light exposure at a wavelength of 365 nm.

#### 4.2.2. Obtaining Composite Gels Loaded with Half-Curcuminoids

The matrices obtained from polysaccharides and loaded with active compounds C1–C4 at a concentration of 0.2% were generated by varying the volumetric ratios of 5% by weight sodium alginate aqueous solution and 2% by weight glucosamine solution in DMSO or water/ethanol solvent mixture (50% by volume), on an ultrasonic bath for 10 min, establishing the optimal ratio of 1:1 (*v*/*v*). After the encapsulation process, the excess of DMSO is removed by washing with water. Unfortunately, a part of the solvent remained retained in the gel network, which could affect the bioactivity of the compounds or have cytotoxic effects [66,67].

### 4.3. Structural Characterization Methods

UV-Vis and fluorescence spectra of the curcumin derivatives in methanol (2 × 10^−4^ mol/L) and gelled materials were recorded with a UV-VIS-NIR Jasco V-570 spectrometer (Jasco Int. Co. Ltd., Tokyo, Japan) at 25 ± 0.5 °C in rectangular quartz cuvettes with an optical path length of 10 mm, in the range 300–600 nm, respectively, and a JASCO FP 6500 spectrofluorimeter (Jasco Int. Co. Ltd., Tokyo, Japan), at an excitation wavelength of 420 nm. The UV-Vis and fluorescence spectra were prepared with Spectra Manager I (Jasco Int. Co. Ltd., Tokyo, Japan).

The IR spectra were recorded in solid dyes and gels on a Jasco FTIR 6300 spectrometer (Jasco Int. Co. Ltd., Tokyo, Japan) equipped with a Specac ATR Golden Gate (Specac Ltd., Orpington, UK). All spectra recorded in the range of 400 to 4000 cm^−1^ (128 accumulations at a resolution of 4 cm^−1^) were processed with Spectra Manager II (Jasco Int. Co. Ltd., Tokyo, Japan).

The synthesized compounds were characterized by gas chromatography coupled with mass spectrometry (GC-MS) with a PERKIN ELMERCLARUS 500 (PerkinElmer Life and Analytical Sciences, Shelton, CT, USA). The gas chromatograph parameters were as follows: injection mode, split; injection volume, 1 µL (at 250 °C); stationary phase, Elite-5MS (5% diphenyl methyl polysiloxane); a 60 m × 0.32 mm × 0.25 µm film thickness of the stationary phase; 70 °C (2 min), 15 °C/min, to 250 °C, 16 min; helium carrier gas; and constant flow = 1 mL/min. The mass spectrometer parameters were as follows: ion source temperature, 230 °C; GC-MS interface temperature, 250 °C; energy electrons, 70 eV; Electronic Impact (EI) ion source in acquisition mode, TIC; and Multiplier, 500 eV. For CHN determination, the FlashSmart Analyzer (Thermo Fisher Scientific, Waltham, MA, USA) was used with the dynamic flash combustion of the sample. The percentage of oxygen was considered as the difference up to the total mass of the analyzed compounds. The samples were placed in tin containers and loaded into the combustion reactor at 950 °C using the Thermo Fisher Scientific™ MAS Plus Autosampler. Following combustion, the resulting analyte gases were carried by a helium flow (140 mL/min) through a copper layer and then passed through a gas chromatography (GC) column for the separation of combustion gases. Subsequently, the gases were detected by a Thermal Conductivity Detector (TCD). The calibration process involved using 2–3 mg of BBOT (2,5-Bis (5-tert-butyl-benzoxazol-2-yl) thiophene) MRC Standard.

1H-NMR and 13C-NMR spectra for the synthesized compounds were registered on a BRUKER AVANCE 400 MHz spectrometer at 25 °C, as solutions in CDCl_3_ and tetramethylsilane as the internal reference. A COSY (Correlation Spectroscopy) experiment was used to identify mutually coupled protons, while an HSQC (Heteronuclear Single Quantum Coherence) experiment was used to determine proton–carbon single bond correlations.

The examination of particle shape and elemental composition was conducted using a scanning electron microscope (SEM) model TM4000Plus from HITACHI in Tokyo, Japan, operated at an accelerating voltage of 15 kV. This SEM was equipped with an energy-dispersive X-ray spectrometer (EDS) model X-stream-2 from Oxford Instruments in Oxford, UK. The elemental composition analysis was carried out using AZtecOne 1.0 software from Oxford Instruments. The weight percentages of copper and all identified elements in organic compounds were determined and reported as weight percentages (wt%) within the specified analysis area.

The stability over time and in relation to the temperature of the composite gels was evaluated by modulated differential scanning calorimetry (MDSC) using an Instrument DSC Q2000 (TA Instruments, New Castle, DE, USA). MTDSC (±0.796 °C/30 s) heat–cool–heat analyses at 10 °C/min were performed in sample pan: Tzero Aluminum, hermetic lid, under helium (99.999%) flow (25 mL/min), as follows: cooling from 30 °C to −80 °C; heating from −80 °C to 30 °C and equilibrating for 3 min for erasing the thermal history, cooling down to −80 °C, isothermal for 3 min, and reheating to 30 °C.

### 4.4. Prediction of the Drug-Likeness and Pharmacokinetic and Pharmacodynamic Features of Compounds

SMILES structures of compounds in both keto and enol forms were obtained based on the 2D structures represented in Marvin [42], as presented in Table S1 (Supplementary Materials). These files were used as input to calculate the pharmacological properties of the compounds. The physicochemical properties of the compounds, as well as their drug-likeness and bioavailability, were predicted using the SwissADME platform [46]. The physicochemical properties that we present include the following: molecular weight (MW), lipophilicity expressed as LogP (the octanol/water partition coefficient), the count of hydrogen bond acceptors (N/O) and donors (NH/OH), and the number of routable bonds (R bonds). The platform estimated the drug-likeness of the compounds by filtering them based on the following rules: (i) Lipinski [45,46]: NH/OH ≤ 5, N/O ≤ 10, MW < 500 Da, LogP < 5; (ii) Veber [47]: R bonds ≤ 10, polar surface area (PSA) < 140 Å^2^; (iii) Ghose [48]: −0.4 < LogP < 5.6, 160 Da < MW < 480 Da, 40 < molar refractivity < 130, 20 < total number of atoms < 70; and (iv) Egan [49]: −0.7 < LogP ≤ 5, PSA ≤ 140 Å^2^ and Muegge [50]: 150 < MW < 500 Da, −2 < LogP < 5, N/O ≤ 10, NH/OH ≤ 5.

The drug distribution properties of the compounds referring to their absorption, distribution, and toxicity were predicted using the pkCSM platform [30]. From all calculated properties, we selected the following to report in this article: Caco2 permeability (parameter to predict the absorption of drugs administered orally at the human intestinal mucosa level), intestinal absorption (the percentage of drug absorbed through the intestine in the case of drugs administered orally), skin permeability (indicative that the drug can be delivered transdermally), BBB permeability of drugs as logBB (reflects the ability of compounds to cross the BBB and be distributed to the CNS), central nervous system permeability expressed as logPS (reflects the ability of drugs to penetrate the CNS), AMES toxicity (the mutagenic potential of the compounds), hERG I and II inhibitor (the ability of the compounds to modulate two important potassium channels that are associated with ventricular arrhythmia), and hepatotoxicity (the ability of compounds to disrupt liver function). The definition of parameters and reference values were considered according to the indications of the pkCSM platform [34].

The pharmacodynamic properties of compounds were predicted using the SwissTarget platform [35] and the Super-PRED Target Prediction platform [44].

### 4.5. Biological Characterization Methods

#### 4.5.1. Cell Culture and Exposure to Half-Curcuminoid Derivatives

**Figure 10:**
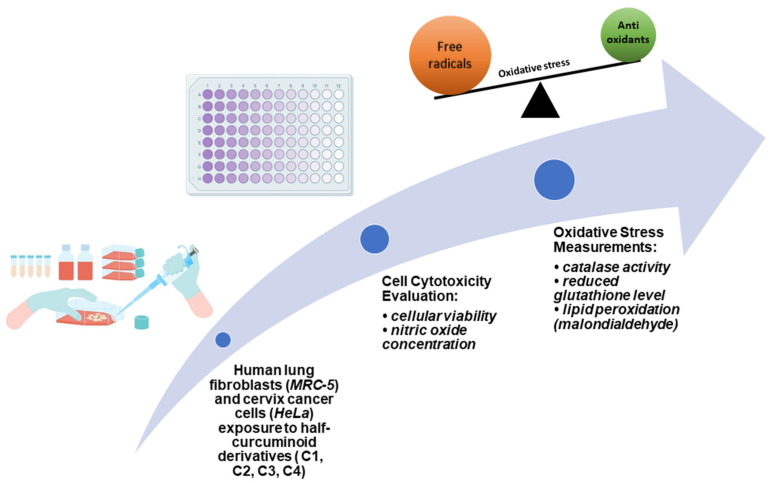
Schematic diagram showing the main biological tests performed on MRC-5 and HeLa cells with the half-curcuminoid derivatives.

The in vitro cytotoxicity of curcumin derivatives was tested using human lung fibroblasts (MRC-5 cell line purchased from American Type Culture Collection (ATCC), Catalog Number CCL-171) and cervix cancer cells (HeLa cell line purchased from ATCC, Catalog Number CCL-2). Both cell lines were grown at 37 °C, in a humidified air with 5% CO_2_, using total Eagle’s minimum essential medium (MEM; Gibco/Invitrogen, Carlsbad, CA, USA) containing 2 mM L-glutamine, 0.1 mM sodium pyruvate, 4.5 g/L glucose, and 10% total bovine serum (FBS; Gibco/Invitrogen, Carlsbad, CA, USA). The growth medium was changed every two days until 80% cell confluence was reached. The cells were then detached and split for subsequent sub-cultivations using a 0.25% (*w*/*v*) Trypsin 0.53 mM EDTA solution (Sigma-Aldrich, St. Louis, MO, USA). After homogenizing 2 mg of powdered curcumin derivative in 50 µL of absolute ethanol, 950 µL of growth medium was added to obtain stock suspensions of 2 mg/mL curcumin derivatives. The resulting suspensions were vortexed and sterilized by UV exposure for 1 h. For biocompatibility assessment and oxidative stress analysis, the cells were seeded on 96-well plates and culture flasks at a density of 1 × 10^4^ cells/cm^2^ and allowed to adhere overnight. After that, the cells were incubated with different concentrations of curcumin derivatives (up to 100 µg/mL) for a maximum of 72 h (Figure 10). For every test, cells cultured in a medium free of curcumin were used as the control group.

#### 4.5.2. Cell Cytotoxicity Assays

The 3-(4,5-dimethylthiazol-2-yl)-2,5-diphenyltetrazolium bromide (MTT; Sigma-Aldrich, MI, USA) colorimetric test, which measures the mitochondrial activity of succinate dehydrogenase in living cells, was used to assess cellular viability. Following 24- and 72-h exposures, the culture medium was replaced with 1 mg/mL MTT solution, and the cells were then incubated for 4 h at 37 °C and 5% CO_2_. The absorbance of the purple formazan crystals formed in the viable cells and dissolved in 2-propanol solution (Sigma-Aldrich, MI, USA) was measured at a wavelength of 595 nm using a microplate reader (GENiosTecan, Grödic, Germany). Since NO plays a significant role in both inflammation and apoptosis, the culture supernatants collected at the end of each incubation period were used to measure the NO concentration as an indicator of cytotoxicity. Briefly, 80 µL culture supernatants were mixed with an equal volume of Griess reagent, a stoichiometric solution (*v*/*v*) of 0.1% naphthylethylenediamine dihydrochloride, and 1% sulphanilamide in 5% H_3_PO_4_. The final absorbance was measured at 550 nm using a microplate reader (GENiosTecan, Grödic, Germany), and the concentration of NO was determined using a NaNO_2_ standard curve.

#### 4.5.3. Biochemical Assays

*Obtaining Cell Lysates and Measurement of Protein Concentration.* In the first step, MRC-5 and HeLa cells were harvested from culture flasks with trypsin, as described in Section 4.5.1, and lysed on ice using ultrasonication (three cycles of 30 s each) (Hielscher UP50H, Teltow, Germany). After centrifuging the cellular homogenates for 10 min at 4 °C and 3000× *g*, the supernatants were utilized for biochemical assays. Using a standard curve of bovine serum albumin and the Bradford reagent (Sigma-Aldrich, St. Louis, MO, USA), the protein concentration of supernatants was determined.

*Antioxidant Enzyme Assay.* CAT activity was assessed by measuring the reduction in absorbance of H_2_O_2_ at 240 nm following Aebi’s method [68]. Under standard conditions, one unit of CAT activity represented the amount of enzyme that could convert 1 µmole of H_2_O_2_ in a minute. The data were collected at pH 7.4 and 25 °C using a SPECORD 200 PLUS double-beam spectrophotometer from AnalytikJena (Jena, Germany). The outcomes were computed as specific enzymatic activities (units/mg of protein) and compared to the control group.

*Reduced Glutathione Assay.* A glutathione assay kit purchased from Sigma-Aldrich (St. Louis, MO, USA) was used to measure the intracellular GSH concentration. Initially, cell lysates were deproteinized with 5% sulfosalicylic acid followed by centrifugation at 4 °C, 10,000× *g* for 10 min. Subsequently, the samples were treated for five minutes at room temperature with 5,5′-dithiobis-2-nitrobenzoic acid (DTNB) in order to transform DTNB into 5-thio-2-nitrobenzoic acid (TNB). Using a microplate reader (TECAN GENios, Grödic, Germany), the optical density at 405 nm was measured, and the GSH content was expressed as nmoles/mg protein in comparison to the control group.

*Lipid Peroxidation Assay.* Malonaldehyde (MDA) can serve as an indicator of lipid peroxidation, with its levels determined through a fluorometric technique. In this process, cell lysates (200 µL) were combined with 700 µL of 0.1 N HCl and left to incubate at room temperature for 20 min. This was followed by the addition of 900 µL of 0.025 M thiobarbituric acid, and then the mixture was then incubated for 65 min at 37 °C. Utilizing the FP-750 spectrofluorometer from Jasco, Tokyo, Japan, the fluorescence units were measured (excitation wavelength = 520 nm; emission wavelength = 549 nm). A 1,1,3,3-tetramethoxypropane standard curve was subsequently used to convert these values to nmoles of MDA. At the end, the MDA concentrations were expressed as nmoles of MDA/mg protein and compared to the control group.

#### 4.5.4. Statistical Analysis

The results were presented as the mean value ± standard deviation (SD) from three separate experiments. Statistical variances between the curcumin derivatives and the control group were assessed using Student’s t-test with GraphPad Prism software (version 5; GraphPad Software, Inc., La Jolla, CA, USA). A *p*-value of less than 0.05 was deemed statistically significant.

## Figures and Tables

**Table 1:** The composition of the mixtures used to generate hydrogels loaded with half-curcuminoid derivatives.

Sample	Alginate H_2_O [mL]	Glucosamine H_2_O/EtOH [mL]	Glucosamine–Dye H_2_O/EtOH [mL]	Glucosamine DMSO [mL]	Glucosamine–Dye DMSO [mL]	Dye EtOH [mL]
G0	1	-	-	1	-	-
						C1
G1	1	1	-	-	-	0.5
G2	1	-	1.5	-	-	-
						C2
G3	1	1	-	-	-	0.5
G4	1	-	-	-	1.5	-
G5	1	-	-	1	-	0.5
G6	1	1 *	-	-	-	0.5
						C3
G7	1	1	-	-	-	0.5
G8	1	-	1.5	-	-	-
						C4
G9	1	-	-	1	-	0.5
G10	1	-	-	-	1.5	-

* Ethanol-free.

**Table 2:** Elemental composition of the hydrogels.

Element	G0 Weight %	G1 Weight %	G2 Weight %	G3 Weight %	G4 Weight %	G5 Weight %	G6 Weight %	G7 Weight %	G8 Weight %	G9 Weight %
C	47.0 (±0.4)	84.5 (±0.2)	51.8 (±0.5)	56.0 (±0.4)	53.8 (±0.4)	50.0 (±0.4)	37.4 (±0.6)	37.3 (±0.9)	30.2 (±1.0)	25.6 (±1.2)
O	36.3 (±0.3)	14.1 (±0.2)	22.4 (±0.3)	24.7 (±0.3)	30.5 (±0.3)	37.7 (±0.3)	33.0 (±0.4)	11.0 (±0.3)	12.4 (±0.3)	11.9 (±0.4)
S	6.1 (±0.1)	0.1 (±0.0)	3.9 (±0.1)	12.3 (±0.1)	5.0 (±0.1)	1.6 (±0.0)	14.9 (±0.2)	1.8 (±0.1)	3.1 (±0.1)	1.1 (±0.0)
Cl	4.9 (±0.1)	1.1 (±0.0)	12.4 (±0.2)	3.3 (±0.0)	5.8 (±0.1)	8.1 (±0.1)	3.1 (±0.1)	27.3 (±0.5)	27.3 (±0.5)	35.4 (±0.7)
N	-	-	-	-	-	-	-	17.0 (±0.8)	18.1 (±0.7)	20.2 (±0.9)
Ca	0.5 (±0.0)	-	1.2 (±0.0)	0.5 (±0.0)	0.6 (±0.1)	0.4 (±0.0)	3.6 (±0.1)	0.6 (±0.0)	-	0.9 (±0.0)
K	0.1 (±0.1)	0.2 (±0.0)	8.0 (±0.1)	3.0 (±0.0)	1.7 (±0.0)	1.9 (±0.0)	8.0 (±0.1)	4.4 (±0.1)	7.9 (±0.1)	4.5 (±0.1)
Na	-	-	0.2 (±0.0)	0.1 (±0.0)	-	0.1 (±0.0)	-	0.2 (±0.0)	0.7 (±0.0)	0.3 (±0.0)
Si	0.1 (±0.0)	-	-	0.1 (±0.0)	0.1 (±0.0)	0.1 (±0.0)	-	0.2 (±0.0)	0.1 (±0.0)	0.1 (±0.0)
Al	4.9 (±0.1)	0.1 (±0.0)	0.1 (±0.0)	0.1 (±0.0)	3.0 (±0.0)	0.1 (±0.0)	0.1 (±0.0)	0.1 (±0.0)	0.3 (±0.0)	0.3 (±0.0)

## Data Availability

The data presented in this study are openly available in article.

## Supplementary Materials

The following supporting information can be downloaded at: https://www.mdpi.com/article/10.3390/gels10060376/s1, Figure S1: SEM images and the EDX spectrum of the alginate; Figure S2. SEM images of the the hydrogel matrix loaded with dyes: C1 (hydrogel G2), C2 (hydrogels G3 and G6), C3 (hydrogels G7 and G8), and C4 (hydrogel G9); Table S1: Predicted physicochemical and drug-likeness properties using SwissADME platform; Table S2. Predicted drug disposition features of the compounds using pkCSM platform; Table S3. SMILES structures of compounds in keto and enol forms.
