# Supplementary material for: Half-Curcuminoids Encapsulated in Alginate–Glucosamine Hydrogel Matrices as Bioactive Delivery Systems
Source: Gels. 2024 May 30;10(6):376. doi: 10.3390/gels10060376 (PMC11203298; doi:10.3390/gels10060376)
Supplement: Supplementary file 1 [file gels-10-00376-s001.zip › gels-2997749-supplementary.pdf]

Supplementary Material

Half-Curcuminoids Encapsulated in Alginate–Glucosamine Hydrogel Matrices as Bioactive Delivery Systems

Florentina Monica Raduly [1], Valentin Raditoiu [1,*], Alina Raditoiu [1], Cristian Andi Nicolae [1], Maria Grapin [1], Miruna Silvia Stan [2,*], Ionela Cristina Voinea [2], Raluca-Ioana Vlasceanu [2], Cristina Doina Nitu [3,4], Dan F. Mihailescu [3], Speranta Avram [3] and Maria Mernea [3]

[1] National Research and Development Institute for Chemistry and Petrochemistry—ICECHIM, 202 Splaiul Independentei, 060021 Bucharest, Romania; monica.raduly@icechim.ro (F.M.R.); coloranti@icechim.ro (A.R.); ca_nicolae@yahoo.com (C.A.N.); maria.grapin@icechim.ro (M.G.)

[2] Department of Biochemistry and Molecular Biology, Faculty of Biology, University of Bucharest, 91-95 Splaiul Independentei, 050095 Bucharest, Romania; cristina.nica@drd.unibuc.ro (I.C.V.); bizon.raluca_ioana@s.bio.unibuc.ro (R.-I.V.)

[3] Department of Anatomy, Animal Physiology and Biophysics, Faculty of Biology, University of Bucharest, 91-95 Splaiul Independentei, 050095 Bucharest, Romania; cristina.nitu@iob.ro (C.D.N.); dan.mihailescu@bio.unibuc.ro (D.F.M.); speranta.avram@bio.unibuc.ro (S.A.); maria.mernea@bio.unibuc.ro (M.M.)

[4] Institute of Oncology "Prof. dr. Al. Trestioreanu", 252, Fundeni, 022328 Bucharest, Romania

* Correspondence: vraditoiu@icechim.ro (V.R.); miruna.stan@bio.unibuc.ro (M.S.S.)

S1. High resolution images of the alginate and hydrogels by SEM analysis

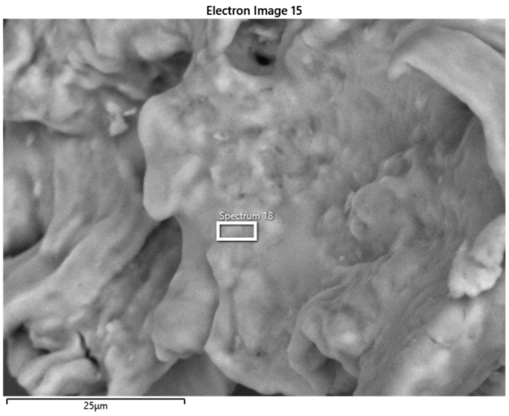

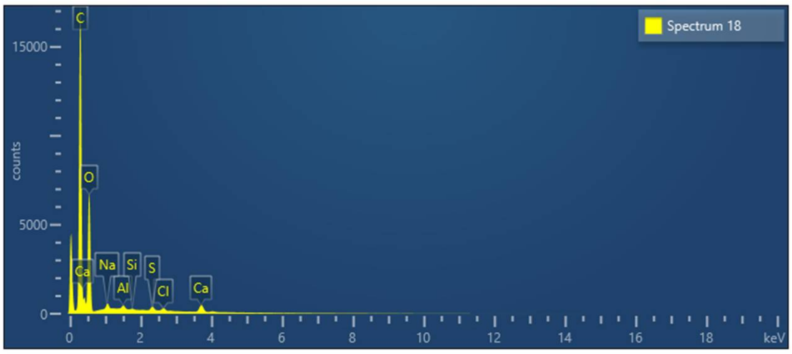

Figure S1. SEM images and the EDX spectrum of the alginate

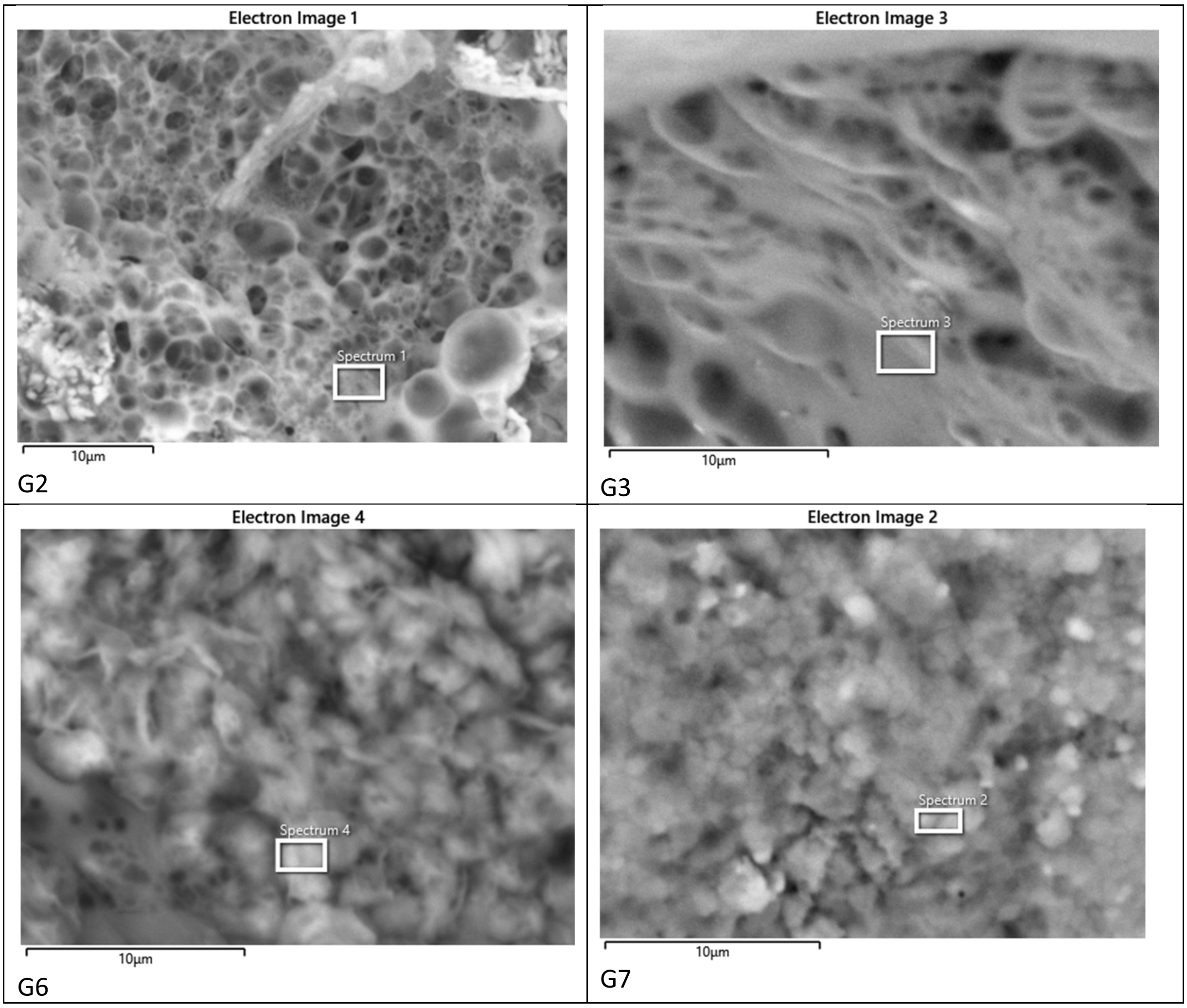

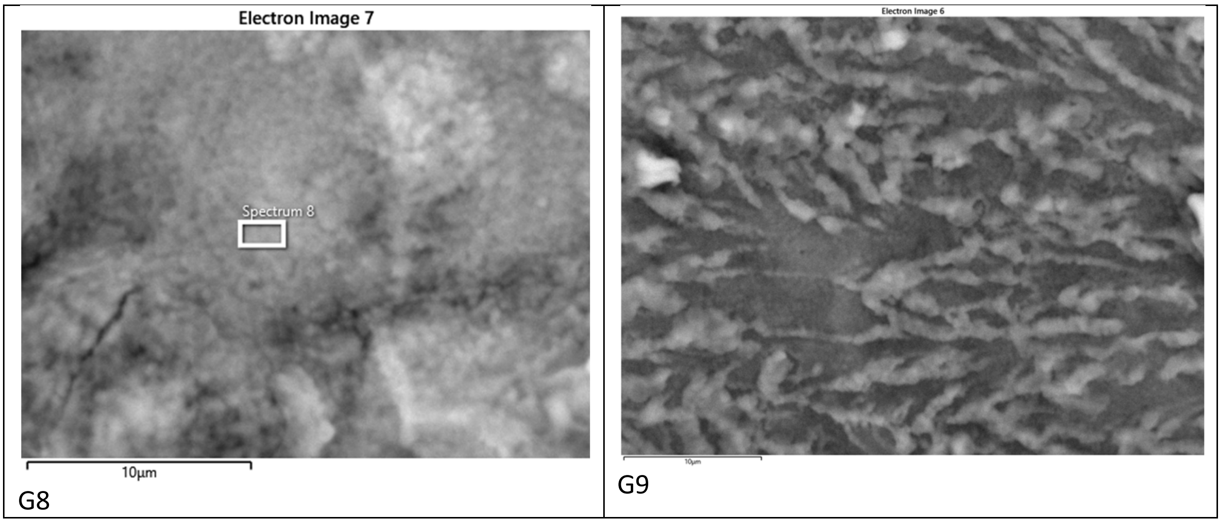

Figure S2. SEM images of the the hydrogel matrix loaded with dyes: C1 (hydrogel G2), C2 (hydrogels G3 and G6), C3 (hydrogels G7 and G8), and C4 (hydrogel G9).

S2. Structural characterization of asymmetric curcumin analogues

The synthesized curcumin-derived compounds were characterized by HPLC, IR, UV-Vis, NMR and MS analyses to confirm their structure and purity. The auxochrome groups grafted onto the aromatic ring influence the photophysical properties of the synthesized compounds, through the interactions established with the environments in which they are found, respectively their polarity which determines the stability of the cis-trans and keto-enol forms of the curcuminic derivatives. The HPLC analyzes confirmed the obtaining of compounds with high purity, following the purification processes C3 having the lowest percentage and requiring additional purification steps. The presence of the two keto-enol isomers is confirmed by the signal at 15 ppm in the ^{1}H-NMR spectra, the singlet chemical shift being characteristic of the O-H bond, the proton forming intramolecular bonds with the two oxygen atoms. At the same time, the structures are confirmed by ^{13}C-NMR, δ value at 180 ppm characteristic of the secondary carbon atom. The stability of the enolic tautomer of the C2 compound is also confirmed in the specialized literature [65]. The chemical shifts around the 7-7.60 ppm value in the ^{1}H-RMN spectra are characteristic of the symmetric double bonds in the aromatic ring and are complemented by the δ values recorded in the range of 148-120 ppm in the ^{13}C-RMN spectra characteristic of tertiary carbons. Hydrogen atoms present in the auxochrome groups are highly descreening and show chemical shifts at δ = 2.17-2.20 ppm for the amide groups, respectively δ = 2.15-1.57 ppm for the methyl groups. Regarding the mass spectra of the compounds studied in our work, it can be observed that the molecular peak ($M^{+\cdot}$} was not the same with the peak base (100%), because at 70 eV in the ionization chamber several fragmentation steps take place from the beginning. Similarities are found, as it was expected, with acetylacetone fragmentation pathway ($M^{+\cdot}$- $CH_3\cdot$; $M^{+\cdot}$- $CH_3CO\cdot$; $M^{+\cdot}$- $CH_3\cdot$ - $CH_3CO\cdot$) and signals of fragmentations belonging to the substituted benzene ring all of them having intensity under 50%. In two cases (C2, C3) is noted the presence of the acetyl fragment, as the base peak. This means the instability of all generated fragments compared to the fragment m/e=43 ($CH_3CO\cdot$). In the other two cases larger fragments have the highest intensity, at m/e=147 which suggests the loss of an isopropenyloxy radical from the molecular structure of the compound C1 and at m/e=244 for C4 showing the stability of the molecular structures probably due to the hydrogen bonds established intramolecularly in the enolic form of the compounds. Anyway, the stability of the molecular radical cation

(from the intensity in the mass spectrum) decreases in the following order C4 (48%) > C2 (45%) > C1 (29%) > C3 (17%).

Table S1. SMILES structures of compounds in keto and enol forms.

Compounds	SMILES structure
C1 keto	CC(=O)CC(=O)\C=C\C1=CC=C(O)C=C1
C1 enol	CC(=O)\C=C(/O)\C=C\C1=CC=C(O)C=C1
C2 keto	COC1=C(O)C=CC(\C=C\C(=O)CC(C)=O)=C1
C2 enol	COC1=C(O)C=CC(\C=C\C(\O)=C\C(C)=O)=C1
C3 keto	CC(=O)CC(=O)\C=C\C1=CC=C(NC(C)=O)C=C1
C3 enol	CC(=O)NC1=CC=C(\C=C\C(\O)=C\C(C)=O)C=C1
C4 keto	CCN(CC)C1=CC=C(\C=C\C(=O)CC(C)=O)C=C1
C4 enol	CCN(CC)C1=CC=C(\C=C\C(\O)=C\C(C)=O)C=C1
G2	CC(CC(=O)\C=C\C1=CC=C(NC(C)=O)C=C1)NC1C(O)OC(CO)C(O)C1O
G8	CC(=O)CC(NC1C(O)OC(CO)C(O)C1O)\C=C\C1=CC=C(O)C=C1

Table S2. Predicted physicochemical and drug-likeness properties using SwissADME platform.

Compound	MW (g/mol)	LogP	N/O	NH/OH	R bonds	Lipinski	Ghose	Veber	Egan	Muegge	B
C1 keto	204.22	1.76	4	3	4	yes	yes	yes	yes	yes	0.55
C1 enol	204.22	1.99	3	2	3	yes	yes	yes	yes	yes	0.85
C2 keto	234.25	1.8	4	1	5	yes	yes	yes	yes	yes	0.55
C2 enol	234.25	2.13	4	2	4	yes	yes	yes	yes	yes	0.85
C3 keto	245.27	1.7	3	1	6	yes	yes	yes	yes	yes	0.55
C3 enol	245.27	2.18	3	2	5	yes	yes	yes	yes	yes	0.85
C4 keto	259.34	2.31	2	0	7	yes	yes	yes	yes	yes	0.55
C4 enol	259.34	3.08	2	1	6	yes	yes	yes	yes	yes	0.85
G2	408.45	0.01	8	6	9	yes	1 violation	1 violation	1 violation	1 violation	0.55
G8	367.39	-0.20	8	6	7	yes	1 violation	yes	1 violation	1 violation	0.55

The calculated properties are: MW – molecular weight, LogP – lipophilicity, N/O – count of hydrogen bond acceptors, NH or OH –count of hydrogen bonds donors, R bonds – count of rotatable bonds, drug-likeness as given by the compliance with Lipinski, Ghose, Veber, Egan and Muegge rules. The predicted Abbot Bioavailability Score is presented in the column denoted by B.

Table S3. Predicted drug disposition features of the compounds using pkCSM platform.

Compound	Caco2 perm. (log cm/s)	Intestinal abs. (%)	Skin permeability (cm/h)	LogBB	LogPS	AMES toxicity	hERG I inhibitor	hERG II inhibitor	hepatotoxicity
C1 keto	1.191	95.623	-2.656	-0.043	-2.428	no	no	no	no
C1 enol	1.203	93.073	-2.883	-0.031	-2.238	no	no	no	no
C2 keto	1.1143	95.459	-2.883	-0.153	-2.526	no	no	no	no
C2 enol	1.137	93.056	-3.029	-0.137	-2.362	no	no	no	no
C3 keto	1.042	95.907	-2.978	-0.052	-2.475	yes	no	no	no
C3 enol	0.941	90.016	-3.383	-0.004	-2.277	no	no	no	no
C4 keto	1.402	96.714	-2.295	0.196	-2.21	no	no	no	no
C4 enol	1.39	92.718	-2.472	0.207	-2.111	no	no	no	no
G2	-0.472	37.294	-2.737	-1.503	-4.161	no	no	no	yes
G8	-0.067	40.127	-2.735	-1.681	-3.915	no	no	no	no

The predicted properties are: (i) Absorption: Caco2 permeability (Caco2 perm.), intestinal absorption (Intestinal abs.), skin permeability, (ii) Distribution: BBB permeability (logBB), CNS permeability (logPS), AMES toxicity, hERG I inhibitor, hERG II inhibitor and hepatotoxicity.
